# Safety and pharmacokinetics of cabazitaxel in patients with hepatic impairment: a phase I dose-escalation study

**DOI:** 10.1007/s00280-016-3210-8

**Published:** 2017-01-05

**Authors:** John Sarantopoulos, Alain C. Mita, Aiwu He, James L. Wade, Chung-Tsen Hsueh, John C. Morris, A. Craig Lockhart, David I. Quinn, Jimmy Hwang, James Mier, Wenping Zhang, Claudine Wack, Jian Yin, Pierre-François Clot, Olivier Rixe

**Affiliations:** 1 Institute for Drug Development, Cancer Therapy and Research Center, University of Texas Health Science Center at San Antonio, San Antonio, TX USA; 2 Samuel Oschin Comprehensive Cancer Institute, Cedars-Sinai Medical Center, Los Angeles, CA USA; 3 Department of Medicine and Oncology and Innovation Center for Biomedical Informatics, Lombardi Comprehensive Cancer Center, Georgetown University, Washington, DC USA; 4 Division of Medical Oncology/Hematology, Cancer Care Center of Decatur, Decatur, IL USA; 5 Division of Medical Oncology/Hematology, Loma Linda University, Loma Linda, CA USA; 6 Division of Hematology-Oncology, Department of Medicine, University of Cincinnati Cancer Institute, Cincinnati, OH USA; 7 Siteman Cancer Center, Washington University School of Medicine, St. Louis, MO USA; 8 University of Southern California Norris Comprehensive Cancer Center, Los Angeles, CA USA; 9 Department of Medicine, Dana-Farber/Harvard Cancer Center, Beth Israel Deaconess Medical Center, Boston, MA USA; 10 Sanofi, Bridgewater, NJ USA; 11 Sanofi, Chilly-Mazarin, France; 12 Division of Hematology/Oncology, Comprehensive Cancer Center, University of New Mexico, Albuquerque, NM USA

**Keywords:** Cabazitaxel, Hepatic impairment, Maximum tolerated dose, Pharmacokinetics, Phase I

## Abstract

**Purpose:**

Cabazitaxel has not been studied in patients with hepatic impairment (HI). This phase I study assessed cabazitaxel safety and pharmacokinetics in patients with HI.

**Methods:**

Patients with advanced, non-hematologic cancer, and normal hepatic function (Cohort 1: C-1), or mild (C-2), moderate (C-3), severe (C-4) HI received cabazitaxel starting doses of 25, 20, 10, and 10 mg/m^2^, respectively. Doses were escalated in patients with HI based on Cycle 1 dose-limiting toxicities (DLTs). Adverse events and the cabazitaxel pharmacokinetic profile were assessed.

**Results:**

In C-2, three patients receiving cabazitaxel 25 mg/m^2^ experienced DLTs; maximum tolerated dose (MTD) was 20 mg/m^2^. In C-3, two patients receiving 20 mg/m^2^ experienced DLTs; MTD was 15 mg/m^2^. C-4 was discontinued early due to DLTs. The most frequent cabazitaxel-related, grade 3–4 toxicity was neutropenia (42%). Cabazitaxel clearance normalized to body surface area (CL/BSA) was lower in C-1 (geometric mean [GM] 13.4 L/h/m^2^) than expected (26.4 L/h/m^2^), but similar in C-2 (23.5 L/h/m^2^) and C-3 (27.9 L/h/m^2^). CL/BSA in C-4 was 18.1 L/h/m^2^. Compared with C-2, CL/BSA increased 19% in C-3 (GM ratio 1.19; 90% CI 0.74–1.91), but decreased 23% in C-4 (0.77; 0.39–1.53). Cabazitaxel free fraction was unaltered. No significant correlation was found between grade 3–4 toxicities and pharmacokinetic parameters.

**Conclusions:**

Mild–moderate HI did not cause substantial decline in cabazitaxel clearance. Cabazitaxel dose reductions in patients with mild–moderate HI, and a contraindication in patients with severe HI, are justified based on safety data.

## Introduction

Cabazitaxel, a second-generation semisynthetic taxane, has demonstrated activity in the second-line treatment of metastatic castration-resistant prostate cancer (mCRPC) after progression on docetaxel-based treatment [1]. Cabazitaxel is approved in combination with prednisone or prednisolone for mCRPC [1–3]. Similar to the first-generation taxanes, paclitaxel and docetaxel, cabazitaxel is primarily metabolized by the liver, mainly by cytochrome P450 CYP3A4/5 isoenzyme and, to a lesser extent, CYP2C8, and is excreted in the bile via the feces [2, 4, 5].

Hepatic impairment may have an unpredictable impact on the pharmacokinetics (PK) of chemotherapies metabolized by the liver, and low serum albumin levels associated with hepatic impairment can result in an increased fraction of free drug leading to increased toxicity [6–9]. Based on this, clinical trials have generally excluded patients with significant hepatic impairment. For many chemotherapy agents, there are no specific data to guide chemotherapy dosing in patients with hepatic impairment and current recommendations remain empiric.

As previous studies of cabazitaxel in solid tumors excluded patients with hepatic impairment, the safety profile of cabazitaxel in this subgroup has not been established [1, 10]. Here, we present the results of a study that examined the PK and safety profile of cabazitaxel in patients with varying degrees of hepatic impairment.

## Materials and methods

### Study design

This was an open-label, dose-escalation, multicenter, phase I study (NCT01140607) of cabazitaxel in patients with non-hematologic cancers and varying degrees of hepatic function. This study was designed to evaluate the maximum tolerated dose (MTD) and safety, and assess the PK properties and relationship between PK and safety parameters, of cabazitaxel in patients with varying degrees of hepatic impairment. A similar design was employed in the study of irinotecan in patients with hepatic dysfunction [11]. This study was approved by ethics committees/review boards at all participating institutions, and all patients provided written informed consent prior to participation. According to the cabazitaxel dose-escalation schedule and dose-escalation decision rules defined in the protocol, which specified different starting dose levels for each cohort and were based on the number of dose-limiting toxicities (DLTs) observed at the different dose levels, a total of 39–75 patients were expected to be enrolled. This sample size would ensure that at least six patients would be enrolled in Cohort 1, 12 patients at MTD in Cohort 2, six patients at MTD in Cohort 3, and six patients at MTD in Cohort 4, in order to evaluate the safety and PK profile of cabazitaxel.

### Patients

Eligible patients were aged ≥18 years with a life expectancy of >3 months, diagnosed with metastatic or locally advanced non-hematologic cancer for which no effective curative therapy was available, had refractory or progressive disease following standard therapies, and had normal hepatic function or chronic hepatic impairment. Patients were enrolled into one of four cohorts based on their degree of hepatic function, defined using National Cancer Institute (NCI) criteria [12]. Cohort 1 had normal hepatic function, defined as total bilirubin and aspartate aminotransferase (AST) ≤ institutional upper limit of normal (ULN); Cohort 2 had mild hepatic impairment, defined as total bilirubin >1.0 to ≤1.5 × ULN or AST >1.5 × ULN; Cohort 3 had moderate hepatic impairment, defined as total bilirubin >1.5 to ≤3.0 × ULN; and Cohort 4 had severe hepatic impairment, defined as total bilirubin >3.0 to 10.0 × ULN. Stable liver function or dysfunction was required. Key exclusion criteria included Eastern Cooperative Oncology Group (ECOG) performance status >2, prior bone marrow transplant or cabazitaxel, known brain metastases, history of Gilbert’s syndrome or grade ≥3 hypersensitivity to taxanes, polysorbate 80 or similar compounds, prior anticancer therapy <3 weeks before study initiation, concurrent or planned participation in another clinical trial, expected need for major surgery or radiation therapy during the study, other concurrent serious illness, acute or chronic medical illness or psychiatric condition that might affect the trial results or the patients’ ability to participate. Patients with significant laboratory abnormalities requiring further investigation, unresolved significant toxicity from prior therapy, or inadequate organ function were also excluded.

### Study treatment

Patients received cabazitaxel during a 1-hour intravenous (IV) infusion on Day 1 of each 3-week cycle until unacceptable toxicity, disease progression, withdrawal of consent, investigator decision, or study cutoff. Different starting doses of cabazitaxel were used for each cohort based on information from the BEX6702 study [13]. The cabazitaxel starting dose (dose level [DL] 0) was based on the level of hepatic function: Cohort 1 (normal function) received 25 mg/m^2^, Cohort 2 (mild hepatic impairment) received 20 mg/m^2^, and Cohorts 3 and 4 (moderate or severe hepatic impairment) received 10 mg/m^2^. The starting dose for Cohort 4 was decided by the Study Committee based on safety and PK findings in the first three patients treated in Cohort 3. Doses were adjusted in Cohorts 2, 3, and 4 based on DLTs observed in Cycle 1. In Cohort 2, doses were adjusted to 15 mg/m^2^ (DL –1) or 25 mg/m^2^ (DL +1), and in Cohorts 3 and 4, doses were adjusted to 15 mg/m^2^ (DL +1), 20 mg/m^2^ (DL +2), or 25 mg/m^2^ (DL +3). If a Cycle 1 DLT was observed in at least two of up to six patients at a given dose level, no further dose escalation occurred. If a Cycle 1 DLT occurred in one of the first three patients treated at a given dose level, three additional patients received that dose. The MTD was the highest dose at which none of the first three patients or one of up to six total patients experienced a Cycle 1 DLT up to the 25 mg/m^2^ dose. Use of granulocyte colony-stimulating factor (G-CSF) was not permitted during Cycle 1.

### Safety assessments

Safety evaluations included vital signs, physical examinations, ECOG performance status, electrocardiograms, and laboratory parameter tests. Adverse events (AEs) were assessed according to the NCI Common Terminology Criteria for AEs (CTCAE) v4.03 [14] from the time of informed consent until ≥30 days after last cabazitaxel dose. DLTs were defined as cabazitaxel-related clinical AEs or laboratory abnormalities. Liver DLTs included increases in bilirubin and/or transaminase levels to three times the baseline value. Other DLTs included grade 3–4 non-hematologic AEs (excluding grade 3 fatigue, anorexia, fever without infection, inadequately treated nausea, vomiting, mucositis or stomatitis; transaminase or bilirubin elevations returning to baseline by next treatment cycle [Cohort 1]; hypersensitivity reaction in the absence of required pre-medication; peripheral neuropathy returning to grade 2 by next treatment cycle) and hematologic toxicity defined as febrile neutropenia, grade 4 neutropenia lasting more than seven days, or grade 4 thrombocytopenia.

### Pharmacokinetic assessments

Heparinized blood samples were collected from all patients in Cycle 1, on Day 1 immediately prior to the start of infusion, 5 min before the end of infusion, and then at 5, 15, and 30 min and 1, 2, 3, 5, 7, and 10 h after the end of infusion. Samples were also collected on Days 2, 3, 4, 5, 8, and 10 after cabazitaxel infusion. Cabazitaxel plasma concentrations were analyzed using a validated liquid chromatography with tandem mass spectrometry method (LC-MS/MS; lower limit of quantitation [LLOQ] = 1 ng/mL). Cabazitaxel PK parameters were calculated using non-compartmental analysis with validated software (PKDMS Version 2.0 with WinNonlin Professional, Version 5.2.1, Pharsight). PK parameters included maximum observed concentration (C_max_), area under the concentration versus time curve extrapolated to infinity (AUC_inf_), area under the concentration versus time curve calculated using the trapezoidal method from time 0 to real-time t_last_ (AUC_last_), terminal half-life (t_1/2z_), total body clearance (CL), and volume of distribution at steady state (V_ss_). CL and V_ss_ were normalized to body surface area (BSA; CL/BSA, V_ss_/BSA). Effect of hepatic impairment on cabazitaxel PK parameters (CL/BSA and dose-normalized exposure parameters [AUC/dose]) was evaluated using linear mixed-effect modelling with degree of hepatic impairment as the fixed effect. Additional plasma samples were collected 5 min before, 3 h after and 24 h after the start of infusion to determine the cabazitaxel free fraction after equilibrium dialysis in buffer using a validated LC-MS/MS method with a LLOQ of 0.1 ng/mL. Cabazitaxel free fraction was estimated using a linear mixed-effect model, with cohort and dose level as fixed effect and time and BSA as continuous variable and patient as random effect.

## Results

### Baseline patient characteristics

Of 77 patients screened, 43 were enrolled including six patients in Cohort 1 (normal hepatic function), 18 in Cohort 2 (mild impairment), 12 in Cohort 3 (moderate impairment), and seven in Cohort 4 (severe impairment) (Table 1). The remaining patients (*n* = 34) were considered non-eligible based on inclusion and/or exclusion criteria. Overall, approximately half of the patients were male, median age was 60 years (range 18–79 years), and most patients (81%) had an ECOG performance status of 1. Patients had various primary tumors with colon and liver the most frequent (19% each). At study entry, most patients had metastatic disease (91%), some had locally advanced disease (7%), and a minority had locoregional recurrence (2%). Median time from cancer diagnosis to first cabazitaxel dose was 2.93 years (range 0.5–17.9 years), and median time from last relapse/progression to first cabazitaxel dose was 1.08 months (range 0.2–24.2 months). Approximately two-thirds of patients had received three or more prior anticancer regimens.

**Table 1:** Baseline patient characteristics

	Cohort 1 25 mg/m^2^ normal hepatic function *n* = 6	Cohort 2 mild hepatic impairment		Cohort 3 moderate hepatic impairment			Cohort 4 severe hepatic impairment			All patients *N* = 43
		20 mg/m^2^ (MTD) *n* = 12	25 mg/m^2^ *n* = 6	10 mg/m^2^ *n* = 3	15 mg/m^2^ (MTD) *n* = 7	20 mg/m^2^ *n* = 2	10 mg/m^2^ *n* = 3	15 mg/m^2^ *n* = 3	20 mg/m^2^ *n* = 1	
Male/female, *n* (%)	3 (50.0)/3 (50.0)	5 (41.7)/7 (58.3)	4 (66.7)/2 (33.3)	3 (100)/0	4 (57.1)/3 (42.9)	0/2 (100)	2 (66.7)/1 (33.3)	0/3 (100)	1 (100)/0	22 (51.2)/21 (48.8)
Age in years, median (range)	68.0 (50–79)	59.0 (18–75)	54.0 (37–70)	64.0 (50–74)	56.0 (52–67)	54.0 (48–60)	64.0 (47–68)	62.0 (38–62)	60.0 (60–60)	60.0 (18–79)
ECOG performance status, *n* (%)										
0	1 (16.7)	1 (8.3)	2 (33.3)	0	1 (14.3)	0	0	0	0	5 (11.6)
1	5 (83.3)	11 (91.7)	4 (66.7)	3 (100)	5 (71.4)	1 (50.0)	2 (66.7)	3 (100)	1 (100)	35 (81.4)
2	0	0	0	0	1 (14.3)	1 (50.0)	1 (33.3)	0	0	3 (7.0)
Months since diagnosis, median (range)	2.91 (1.0–17.9)	3.02 (0.5–17.2)	3.61 (1.1–8.8)	6.17 (1.5–8.7)	2.38 (1.6–3.7)	2.16 (0.7–3.6)	1.38 (0.8–14.1)	3.34 (1.8–4.9)^a^	2.99 (3.0–3.0)	2.93 (0.5–17.9)^b^
Primary tumor site, *n* (%)										
Breast	2 (33.3)	1 (8.3)	0	0	1 (14.3)	0	0	1 (33.3)	0	5 (11.6)
Colon	0	2 (16.7)	0	1 (33.3)	1 (14.3)	1 (50.0)	1 (33.3)	1 (33.3)	1 (100)	8 (18.6)
Head/neck	0	0	0	0	0	0	1 (33.3)	0	0	1 (2.3)
Liver	0	3 (25.0)	1 (16.7)	0	4 (57.1)	0	0	0	0	8 (18.6)
Lungs	2 (33.3)	0	1 (16.7)	0	0	1 (50.0)	0	0	0	4 (9.3)
Pancreas	1 (16.7)	0	0	0	0	0	0	0	0	1 (2.3)
Prostate	0	2 (16.7)	0	1(33.3)	0	0	0	0	0	3 (7.0)
Other^c^	1 (16.7)	4 (33.3)	4 (66.7)	1 (33.3)	1 (14.3)	0	1 (33.3)	1 (33.3)	0	13 (30.2)
Disease extent at study entry, *n* (%)										
Locally advanced	0	0	0	0	3 (42.9)	0	0	0	0	3 (7.0)
Locoregional recurrence	0	0	0	0	1 (14.3)	0	0	0	0	1 (2.3)
Metastatic	6 (100)	12 (100)	6 (100)	3 (100)	3 (42.9)	2 (100)	3 (100)	3 (100)	1 (100)	39 (90.7)
Prior anticancer regimens, *n* (%)										
1	0	1 (8.3)	2 (33.3)	0	2 (28.6)	1 (50.0)	0	0	0	6 (14.0)
2	2 (33.3)	4 (33.3)	0	0	1 (14.3)	0	0	2 (66.7)	0	9 (20.9)
≥3	4 (66.7)	7 (58.3)	4 (66.7)	3 (100)	4 (57.1)	1 (50.0)	3 (100)	1 (33.3)	1 (100)	28 (65.1)

*ECOG* Eastern Cooperative Oncology Group; *MTD* maximum tolerated dose

^a^ *n* = 2

^b^ *n* = 42

^c^basal cell carcinoma (skin), bladder cancer, cholangiocarcinoma, esophageal cancer, gastroesophageal junction, lower right anterior chest synovial cell sarcoma, mesothelioma, neuroendocrine carcinoma of the ileocecal valve, pancreatic neuroendocrine carcinoma, rectal cancer

### Treatment

The cabazitaxel doses administered in each cohort were as follows: Cohort 1 (normal hepatic function), 25 mg/m^2^; Cohort 2 (mild impairment), 20 and 25 mg/m^2^; and Cohorts 3 (moderate impairment) and 4 (severe impairment), 10, 15, and 20 mg/m^2^ (Table 1). Patients received a median of two cycles of cabazitaxel (range 1–31 cycles) (Table 2). The median number of cycles and relative dose intensity versus planned dose was similar across cohorts. The median duration of treatment was 6 weeks (range 3–107 weeks). All patients had discontinued study treatment at study cutoff, except for one patient in Cohort 2 (20 mg/m^2^) who had received 31 cycles. Primary reasons for cabazitaxel discontinuation included disease progression (65%) and toxicity/AEs (21%) (Table 2).

**Table 2:** Treatment characteristics

	Cohort 1 25 mg/m^2^ normal hepatic function *n* = 6	Cohort 2 mild hepatic impairment		Cohort 3 moderate hepatic impairment			Cohort 4 severe hepatic impairment			All patients *N* = 43
		20 mg/m^2^ (MTD) *n* = 12	25 mg/m^2^ *n* = 6	10 mg/m^2^ *n* = 3	15 mg/m^2^ (MTD) *n* = 7	20 mg/m^2^ *n* = 2	10 mg/m^2^ *n* = 3	15 mg/m^2^ *n* = 3	20 mg/m^2^ *n* = 1	
Total cabazitaxel cycles by patient, *n*	16	54	12	6	13	2	6	8	1	118
Median caba zitaxel cycles by patient, *n* (range)	3 (1–4)	2 (1–31)	1 (1–6)	1 (1–4)	2 (1–3)	1 (1–1)	1 (1–4)	3 (1–4)	1 (1–1)	2 (1–31)
Relative dose intensity, mg/m^2^/week, median (range)	0.99 (0.63–1.01)	0.97 (0.84–1.02)	1.01 (0.70–1.02)	0.99 (0.81–1.01)	0.90 (0.72–1.01)	1.03 (1.00–1.05)	1.00 (0.92–1.02)	1.01 (0.88–1.05)	0.99 (0.99–0.99)	0.99 (0.63–1.05)
Weeks of study treatment, median (range)	9.07 (3.0–16.1)	6.14 (3.0–107.0)	3.00 (3.0–20.9)	3.00 (3.0–12.1)	6.00 (3.0–10.0)	3.00 (3.0–3.0)	3.00 (3.0–13.1)	10.29 (3.0–12.0)	3.00 (3.0–3.0)	6.00 (3.0–107.0)
Treatment discontinuation, *n* (%)	6 (100)	11 (91.7)	6 (100)	3 (100)	7 (100)	2 (100)	3 (100)	3 (100)	1 (100)	42 (97.7)
Adverse event	1 (16.7)	2 (16.7)	1 (16.7)	1 (33.3)	3 (42.9)	0	0	0	1 (100)	9 (20.9)
Disease progression	4 (66.7)	8 (66.7)	4 (66.7)	2 (66.7)	4 (57.1)	2 (100)	3 (100)	1 (33.3)	0	28 (65.1)
Other reason	1 (16.7)	1 (8.3)	1 (16.7)	0	0	0	0	2 (66.7)	0	5 (11.6)

*MTD* maximum tolerated dose

### Safety data

Of the 43 treated patients, 38 were evaluable for DLTs with five excluded due to concomitant G-CSF administration during Cycle 1 in the absence of a DLT. In Cycle 1, 13 patients (34%) across all cohorts experienced a DLT (Table 3). Hematologic and non-hematologic DLTs were each reported in eight patients (21%) with three patients experiencing both. No liver-related DLTs were reported. In Cohort 2 (mild hepatic impairment), three of five patients receiving 25 mg/m^2^ experienced DLTs (grade 4 febrile neutropenia, grade 3 hypophosphatemia, and grade 4 neutropenia without fever), and the MTD was established as 20 mg/m^2^. In Cohort 3 (moderate impairment), the first two patients treated at 20 mg/m^2^ experienced DLTs (grade 4 neutropenic sepsis, and grade 3 febrile neutropenia and stoma site infection), and the MTD was established as 15 mg/m^2^. In Cohort 4 (severe impairment), the MTD was not established because no patient treated with 10 or 15 mg/m^2^ experienced DLTs during Cycle 1 and treatment was discontinued early for this cohort because the first patient treated at 20 mg/m^2^ experienced DLTs and subsequently died from a combination of septic shock, tumor lysis syndrome and acute respiratory failure in the context of acute renal failure and disease progression. Based on this outcome, patient accrual into Cohort 4 was discontinued.

**Table 3:** Dose-limiting toxicities and treatment-emergent adverse events

	Cohort 1 25 mg/m^2^ normal hepatic function *n* = 6	Cohort 2 mild hepatic impairment		Cohort 3 moderate hepatic impairment			Cohort 4 severe hepatic impairment			All patients *N* = 43
		20 mg/m^2^ (MTD) *n* = 12	25 mg/m^2^ *n* = 6	10 mg/m^2^ *n* = 3	15 mg/m^2^ (MTD) *n* = 7	20 mg/m^2^ *n* = 2	10 mg/m^2^ *n* = 3	15 mg/m^2^ *n* = 3	20 mg/m^2^ *n* = 1	
	*n* = 4	*n* = 11	*n* = 5	*n* = 3	*n* = 6	*n* = 2	*n* = 3	*n* = 3	*n* = 1	*N* = 38^a^
DLT during Cycle 1, *n* (%)	3 (75.0)	3 (27.3)	3 (60.0)	0	1 (16.7)	2 (100)	0	0	1 (100)	13 (34.2)
Hematologic	2 (50.0)	3 (27.3)	2 (40.0)	0	0	1 (50.0)	0	0	0	8 (21.1)
Non-hematologic	1 (25.0)	2 (18.2)	1 (20.0)	0	1 (16.7)	2 (100)	0	0	1 (100)	8 (21.1)
Liver	0	0	0	0	0	0	0	0	0	0
Grade 3–4 TEAE, *n* (%)	6 (100)	11 (91.7)	6 (100)	3 (100)	6 (85.7)	2 (100)	3 (100)	2 (66.7)	1 (100)	40 (93.0)
Grade 3–4 treatment-related TEAE, *n* (%)	5 (83.3)	9 (75.0)	6 (100)	1 (33.3)	6 (85.7)	2 (100)	1 (33.3)	1 (33.3)	1 (100)	32 (74.4)
Grade 3–4 treatment-related TEAEs occurring in >1 patient, *n* (%)										
Neutropenia	5 (83.3)	3 (25.0)	4 (66.7)	1 (33.3)	4 (57.1)	0	0	1 (33.3)	0	18 (41.9)
Febrile neutropenia	1 (16.7)	3 (25.0)	1 (16.7)	0	1 (14.3)	1 (50.0)	0	0	0	7 (16.3)
Anemia	1 (16.7)	2 (16.7)	0	0	0	0	0	1 (33.3)	1 (100)	5 (11.6)
Leukopenia	0	2 (16.7)	0	0	2 (28.6)	0	0	0	0	4 (9.3)
Fatigue	2 (33.3)	0	0	0	0	1 (50.0)	0	0	0	3 (7.0)
Lymphopenia	1 (16.7)	1 (8.3)	1 (16.7)	0	0	0	0	0	0	3 (7.0)
Diarrhea	0	1 (8.3)	0	0	0	0	0	1 (33.3)	0	2 (4.7)
Dehydration	0	1 (8.3)	0	0	0	0	0	0	1 (100)	2 (4.7)
Urinary tract infection	0	0	0	0	0	0	0	1 (33.3)	1 (100)	2 (4.7)
Platelet count decreased	0	2 (16.7)	0	0	0	0	0	0	0	2 (4.7)
White blood cell count decreased	0	1 (8.3)	0	0	0	1 (50.0)	0	0	0	2 (4.7)
Any grade, any causality TEAE leading to discontinuation, *n* (%)	1 (16.7)	2 (16.7)	1 (16.7)	1 (33.3)	3 (42.9)	0	0	0	1 (100)	9 (20.9)
Any grade, any causality hepatobil i aryTEAE, *n* (%)	0	0	1 (16.7)	1 (33.3)	4 (57.1)	0	0	0	0	6 (14.0)
Hepatic failure	0	0	1 (16.7)	0	1 (14.3)	0	0	0	0	2 (4.7)
Jaundice	0	0	0	1 (33.3)	1 (14.3)	0	0	0	0	2 (4.7)
Cholangitis	0	0	0	0	1 (14.3)	0	0	0	0	1 (2.3)
Hyperbilirubinemia	0	0	0	0	1 (14.3)	0	0	0	0	1 (2.3)

*DLT* dose-limiting toxicity; *MTD* maximum tolerated dose; *TEAE* treatment-emergent adverse event

^a^DLT-evaluable population (*N* = 38): five patients excluded due to the administration of granulocyte colony-stimulating factor during the first three weeks of treatment in the absence of a DLT

AEs were assessed in all patients (Table 3). The most frequent treatment-emergent AEs (TEAEs) of any grade (in >25% of patients overall), regardless of causality, were fatigue (54%), neutropenia (42%), diarrhea (40%), nausea (40%), anemia (37%), vomiting (35%), abdominal pain (28%), and peripheral edema (26%). The most frequent TEAEs were observed in all cohorts, except for vomiting which was not reported in Cohort 1. The most frequent grade 3–4 TEAEs (in >3 patients overall), regardless of causality, were neutropenia (42%), anemia (23%), febrile neutropenia (16%), abdominal pain (14%), leukopenia (9%), and dehydration (9%). Six patients (14%) presented with a TEAE (of any causality) related to hepatobiliary disorders: one patient in Cohort 2 (mild hepatic impairment) and five patients in Cohort 3 (moderate impairment). Neutropenia was the most frequent grade 3–4 treatment-related TEAE (Table 3). Analysis of AEs did not reveal any trends related to hepatic impairment.

### Pharmacokinetics

**Fig. 1:**
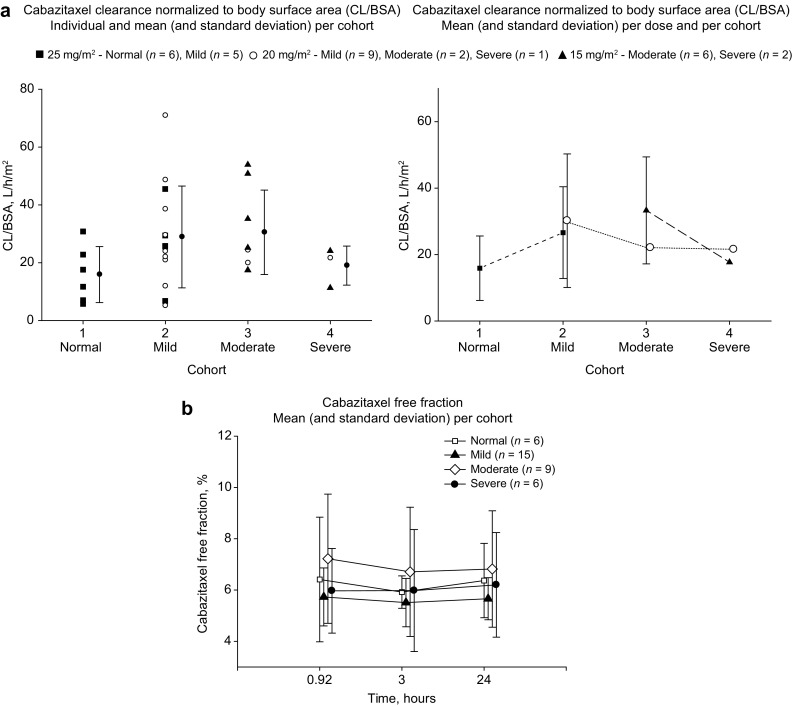
Pharmacokinetic analysis in the pharmacokinetic population (erratic profiles excluded) of **a** CL/BSA **b** cabazitaxel free fraction

Of 43 patients, 38 were eligible for PK assessment (Table 4). Four patients were excluded because of PK deviations and one patient because of ineligibility for any defined cohort in the study. In addition, two patients from Cohort 3 receiving cabazitaxel 10 mg/m^2^ were excluded from PK analysis because they displayed aberrant PK behaviors, including a very low C_max_ and a mean CL/BSA (517 L/h/m^2^) that was approximately 20-fold higher than other patients in Cohort 3 (30.5 L/h/m^2^ for patients receiving cabazitaxel 15 and 20 mg/m^2^ collectively). The CL/BSA estimate for patients in Cohort 1 (normal hepatic function; 13.4 L/h/m^2^) was in the very low range of typical cabazitaxel clearance shown in a previous population PK analysis (26.4 L/h/m^2^, coefficient of variation: 38.8%; *n* = 170) [15] and other phase I studies assessing cabazitaxel PK (28.6 L/h/m^2^, *n* = 4 [13]; 27.3 L/h/m^2^, *n* = 25 [16]; 44.7 L/h/m^2^ *n* = 21 [17]). Because of this unusually low cabazitaxel clearance in Cohort 1, meaningful PK comparisons could not be made between patients with hepatic impairment and normal hepatic function. As a result, comparisons were made using patients with mild hepatic impairment. Compared with Cohort 2 (mild hepatic impairment), Cohort 3 (moderate impairment) showed a 19% increase in CL/BSA, associated with a 14% decrease in AUC_last_/dose, whereas Cohort 4 (severe impairment) showed a 23% decrease in CL/BSA, associated with a 17% increase in AUC_last_/dose (Table 5; Fig. 1a). A sensitivity analysis, which excluded patients with erratic PK profiles, showed consistent findings to the main analysis. Compared with Cohort 2, Cohort 3 showed a 6% decrease in CL/BSA (ratio: 0.94; 90% CI 0.64–1.38) and Cohort 4 showed a 39% decrease (ratio: 0.61; 90% CI 0.36–1.05). Hepatic impairment did not affect the free fraction of cabazitaxel (5.6–6.6% across the cohorts); thus, analysis of free drug PK led to the same conclusions as for total drug PK (Fig. 1b).

**Table 4:** Pharmacokinetic parameters: descriptive statistics

Parameter: mean ± SD (geometric mean) [CV%]	Cohort 1 25 mg/m^2^ normal hepatic function *n* = 6	Cohort 2 mild hepatic impairment		Cohort 3 moderate hepatic impairment			Cohort 4 severe hepatic impairment		
		20 mg/m^2^ (MTD) *n* = 9	25 mg/m^2^ *n* = 6	10 mg/m^2^ * n* = 2^a^	15 mg/m^2^ (MTD) *n* = 7	20 mg/m^2^ *n* = 2	10 mg/m^2^ *n* = 2	15 mg/m^2^ *n* = 3	20 mg/m^2^ *n* = 1
C_max_, ng/mL	691 ± 563 (545) [82]	294 ± 253 (213) [86]	328 ± 189 (287) [57]	16.4 ± NC (15.4) [NC]	212 ± 275 (131) [130]	333 ± NC (294) [NC]	378 ± NC (207) [NC]	117 ± 93.9 (92.7) [81]	77.4 ± NC (77.4) [NC]
AUC_last_, ng*h/mL	2010 ± 1420 (1610) [70]	893 ± 1000 (612) [112]	1040 ± 1050 (787) [100]	184 ± NC (54.0) [NC]	387 ± 154 (360) [40]	684 ± NC (674) [NC]	998 ± NC (542) [NC]	585 ± 507 (454) [87]	591 ± NC (591) [NC]
t_1/2z_, h	77.9 ± 36.9 (69.7) [47]	90.4 ± 38.8 (81.0) [43]	94.7 ± 12.0 (94.1) [13]	3.47 ± NC (2.27) [NC]	98.4 ± 62.3 (78.6) [63]	111 ± NC (110) [NC]	76.3 ± NC (76.2) [NC]	111 ± 5.23 (111) [5]	141 ± NC (141) [NC]
AUC_inf_, ng*h/mL	2220 ± 1410 (1860) [63]	1110 ± 1090 (829) [98]	1420 ± 1300 (1110) [92]^b^	206 ± NC (62.8) [NC]	526 ± 243 (478) [46]^c^	931 ± NC (925) [NC]	NC ± NC (NC) [NC]^d^	974 ± NC (909) [NC]^e^	915 ± NC (915) [NC]
CL, L/h	27.6 ± 16.7 (23.1) [60]	55.0 ± 38.5 (44.0) [70]	46.9 ± 25.4 (40.0) [54]^b^	970 ± NC (314) [NC]	62.5 ± 33.0 (56.1) [53]^c^	39.6 ± NC (39.4) [NC]	NC ± NC (NC) [NC]^d^	33.4 ± NC (31.7) [NC]^e^	36.7 ± NC (36.7) [NC]
V_ss_, L	2220 ± 2450 (1410) [110]	4940 ± 2440 (3820) [49]	4040 ± 1630 (3740) [40]^b^	2580 ± NC (2440) [NC]	4800 ± 3160 (3950) [66]^c^	4660 ± NC (4410) [NC]	NC ± NC (NC) [NC]^d^	5330 ± NC (4170) [NC]^e^	7230 ± NC (7230) [NC]
CL/BSA, L/h/m^2^	15.9 ± 9.70 (13.4) [61]	30.2 ± 20.1 (24.0) [67]	26.6 ± 13.8 (22.7) [52]^b^	517 ± NC (143) [NC]	33.3 ± 16.1 (30.1) [48]^c^	22.1 ± NC (22.0) [NC]	NC ± NC (NC) [NC]^d^	17.7 ± NC (16.5) [NC]^e^	21.6 ± NC (21.6) [NC]
V_ss_/BSA, L/m^2^	1250 ± 1300 (820) [104]	2770 ± 1440 (2080) [52]	2310 ± 924 (2120) [40]^b^	1130 ± NC (1110) [NC]	2380 ± 1130 (2120) [47]^c^	2610 ± NC (2460) [NC]	NC ± NC (NC) [NC]^d^	2860 ± NC (2180) [NC]^e^	4250 ± NC (4250) [NC]

*AUC*_*inf*_ area under the plasma concentration–time curve extrapolated to infinity; *AUC*_*EXT*_ extrapolated area under the plasma concentration–time curve; *AUC*_*last*_ area under the plasma concentration–time curve from time zero to the time of the last cabazitaxel concentration; *CL* clearance; *CL/BSA* clearance normalized to body surface area; *C*_*max*_ maximum observed plasma concentration; *CV* coefficient of variation; *NC* not calculated; *SD* standard deviation; *t*_*1/2z*_ apparent terminal half-life; *V*_*ss*_ volume of distribution at steady state; *V*_*ss*_ */BSA* volume of distribution at steady state normalized to body surface area

^a^Patients in Cohort 3 receiving cabazitaxel 10 mg/m^2^ and displaying aberrant PK behaviors (very low C_max_) were excluded from the statistical analysis

^b^ *n* = 5, parameter not calculable for one patient (AUC_Ext_ >40%)

^c^ *n* = 6, parameter not calculable for one patient (AUC_Ext_ >40%)

^d^ *n* = 0, parameter not calculable for two patients (AUC_Ext_ >40%)

^e^ *n* = 2, parameter not calculable for one patient (AUC_Ext_ >40%)

**Table 5:** Pharmacokinetic parameters: effect of hepatic impairment

Parameter	Cohort (hepatic function/impairment)	*n*	Geometric mean (90% CI)	Versus cohort 1 (normal hepatic function) Ratio (90% CI)	Versus cohort 2 (mild hepatic impairment) Ratio (90% CI)
CL, L/h	Cohort 1 (normal)	6	23.00 (14.73, 35.92)	1.00	–
	Cohort 2 (mild)^a^	14	42.54 (31.92, 56.71)	1.85 (1.09, 3.14)	–
	Cohort 3 (moderate)^b^	8	51.50 (35.12, 75.53)	2.24 (1.24, 4.05)	1.21 (0.75, 1.95)
	Cohort 4 (severe)^c^	3	33.32 (17.91, 62.01)	1.45 (0.67, 3.12)	0.78 (0.40, 1.55)
CL/BSA, L/h/m^2^	Cohort 1 (normal)	6	13.42 (8.64, 20.83)	1.00	–
	Cohort 2 (mild)^a^	14	23.51 (17.63, 31.35)	1.75 (1.04, 2.96)	–
	Cohort 3 (moderate)^b^	8	27.86 (19.03, 40.77)	2.08 (1.16, 3.72)	1.19 (0.74, 1.91)
	Cohort 4 (severe)^c^	3	18.13 (9.73, 33.76)	1.35 (0.63, 2.89)	0.77 (0.39, 1.53)
AUC_inf_/dose, ng*h/mL/mg/m^2^	Cohort 1 (normal)	6	74.52 (48.01, 115.68)	1.00	–
	Cohort 2 (mild)^a^	14	42.53 (31.89, 56.72)	0.57 (0.34, 0.97)	–
	Cohort 3 (moderate)^b^	8	35.91 (24.53, 52.55)	0.48 (0.27, 0.86)	0.84 (0.52, 1.36)
	Cohort 4 (severe)^c^	3	55.17 (29.62, 102.75)	0.74 (0.35, 1.59)	1.30 (0.65, 2.57)
AUC_last_/dose, ng*h/mL/mg/m^2^	Cohort 1 (normal)	6	64.21 (38.39, 107.40)	1.00	–
	Cohort 2 (mild)	15	31.09 (22.46, 43.05)	0.48 (0.26, 0.89)	–
	Cohort 3 (moderate)^d^	9	26.6 (17.48, 40.48)	0.41 (0.21, 0.80)	0.86 (0.50, 1.46)
	Cohort 4 (severe)	6	36.24 (21.67, 60.62)	0.56 (0.27, 1.17)	1.17 (0.63, 2.14)
C_max_/dose, ng/mL/mg/m^2^	Cohort 1 (normal)	6	21.78 (12.11, 39.18)	1.00	–
	Cohort 2 (mild)	15	11.01 (7.60, 15.97)	0.51 (0.25, 1.01)	–
	Cohort 3 (moderate)^d^	9	10.08 (6.24, 16.28)	0.46 (0.22, 0.99)	0.92 (0.50, 1.68)
	Cohort 4 (severe)	6	8.46 (4.71, 15.23)	0.39 (0.17, 0.89)	0.77 (0.38, 1.54)
t_1/2z_, h	Cohort 1 (normal)	6	71.07 (49.36, 102.31)	1.00	–
	Cohort 2 (mild)	15	85.92 (68.55, 107.69)	1.21 (0.79, 1.86)	–
	Cohort 3 (moderate)^d^	9	83.64 (62.30, 112.29)	1.18 (0.73, 1.89)	0.97 (0.67, 1.41)
	Cohort 4 (severe)	6	102.12 (71.46, 145.92)	1.44 (0.86, 2.39)	1.19 (0.78, 1.81)
V_ss_, L	Cohort 1 (normal)	6	1442.95 (802.87, 2593.34)	1.00	–
	Cohort 2 (mild)^a^	14	3785.17 (2593.95, 5523.44)	2.62 (1.31, 5.27)	–
	Cohort 3 (moderate)^b^	8	4005.9 (2421.05, 6628.21)	2.78 (1.27, 6.06)	1.06 (0.56, 1.99)
	Cohort 4 (severe)^c^	3	4981.61 (2201.05, 11,274.82)	3.45 (1.26, 9.46)	1.32 (0.54, 3.24)
V_ss_/BSA, L/m^2^	Cohort 1 (normal)	6	819.68 (464.42, 1446.71)	1.00	–
	Cohort 2 (mild)^a^	14	2093.46 (1443.24, 3036.63)	2.55 (1.30, 5.04)	–
	Cohort 3 (moderate)^b^	8	2201.19 (1345.79, 3600.29)	2.69 (1.27, 5.69)	1.05 (0.57, 1.95)
	Cohort 4 (severe)^c^	3	2729.48 (1222.20, 6095.62)	3.33 (1.24, 8.91)	1.30 (0.54, 3.16)

AUC_inf_/dose, area under the plasma concentration–time curve extrapolated to infinity normalized to dose; AUC_EXT_, extrapolated area under the plasma concentration–time curve; AUC_last_/dose, area under the plasma concentration–time curve from time zero to the time of the last cabazitaxel concentration, normalized to dose; CL, clearance; CL/BSA, clearance normalized to body surface area; CI, confidence interval; C_max_/dose, maximum observed plasma concentration normalized to dose; t_1/2z_, apparent terminal half-life; V_ss_, volume of distribution at steady state; V_ss_/BSA, volume of distribution at steady state normalized to body surface area

^a^ *n* = 14, parameter not calculable for one patient (AUC_Ext_ >40%)

^b^ *n* = 8, patients receiving cabazitaxel 10 mg/m^2^ and displaying aberrant PK behaviors (very low C_max_) were excluded from the statistical analysis and parameter not calculable for one patient (AUC_Ext_ > 40%)

^c^ *n* = 3, parameter not calculable for three patients (AUC_Ext_ > 40%)

^d^ *n* = 9, patients receiving cabazitaxel 10 mg/m^2^ and displaying aberrant PK behaviors (very low C_max_) were excluded from the statistical analysis

### Correlation between safety and PK parameters

No significant correlation (*p* < 0.05) was found between grade 3–4 TEAEs or laboratory abnormalities in Cycle 1 and PK parameters. Study cohort (degree of hepatic function) was not a statistically significant covariate in any of the logistic regression models of PK parameters.

### Efficacy

There were no efficacy endpoints in this study, and therefore, data were not routinely collected. However, one patient with cholangiocarcinoma had stable disease, which was maintained at Cycle 32.

## Discussion

This study assessed the safety and PK of cabazitaxel in patients with hepatic impairment compared with patients who have normal hepatic function. Cabazitaxel is primarily metabolized by the liver, and therefore, it is important to assess the effect of hepatic impairment on cabazitaxel metabolism. The first-generation taxanes, docetaxel and paclitaxel, are administered at lower doses in patients with hepatic impairment because of an increased risk of myelosuppression, stomatitis, neutropenia, and treatment-related death [4, 5, 18, 19].

In this study, parameters used for patient recruitment and for defining hepatic impairment levels were based on the NCI criteria [12] and were previously used in a study assessing irinotecan in patients with hepatic dysfunction [11]. These parameters made patient recruitment challenging, particularly for severely impaired patients. Using albumin levels or Child-Pugh-Turcotte classification scores, versus metabolic status, to define hepatic function and guide cohort allocation may have been beneficial and may have provided a more accurate characterization of hepatic function. Potentially, albumin levels could have been correlated with PK and safety parameters.

The MTD of cabazitaxel administered by IV infusion every 3 weeks in patients with advanced solid tumors was determined to be 20 and 15 mg/m^2^ for patients with mild or moderate hepatic impairment, respectively. For patients with severe hepatic impairment, treatment in Cohort 4 was prematurely discontinued following the death of the first patient treated with cabazitaxel 20 mg/m^2^ from a combination of AEs (including DLTs) and disease progression; therefore, the MTD in this cohort of patients remains undetermined.

The overall safety profile of cabazitaxel was consistent with the known safety profile with no new safety issues identified. The safety profile of cabazitaxel 20 and 15 mg/m^2^ (MTD) in patients with mild or moderate hepatic impairment, respectively, was generally similar to that observed in patients with normal hepatic function receiving 25 mg/m^2^. In this study, prophylactic use of G-CSF was not permitted during Cycle 1. Prophylactic administration of G-CSF has the ability to reduce hematologic toxicity in clinical practice.

As CL/BSA for cabazitaxel in patients with normal hepatic function was low compared with historical data [13, 15–17], PK data for patients with normal hepatic function in this study could not be used in comparisons. The reason for these low values is unclear; patients with no reason for exclusion showed variability in parameters and erratic PK profiles. The number of patients with normal hepatic function was small (*n* = 6), which may help explain the large variability in cabazitaxel clearance for this cohort as several outliers considerably affected the average values of the cohort and created a substantial shift in average CL/BSA. Patients with mild or moderate hepatic impairment had CL/BSA values comparable to historical data, suggesting no influence of mild or moderate hepatic impairment on cabazitaxel PK. There was no evidence that moderate versus mild hepatic impairment resulted in a substantial decline in cabazitaxel clearance. There was no evidence that the lower MTD in patients with mild or moderate hepatic impairment, compared with the approved 25 mg/m^2^ cabazitaxel dose, were due to higher cabazitaxel exposure. Patients with severe hepatic impairment had a numerically decreased CL/BSA compared with mildly impaired patients, indicating some effect of severe impairment on PK parameters. This numerical increase in cabazitaxel exposure may, in part, explain the increased toxicity of cabazitaxel observed in this patient cohort. However, because of the limitations of a small study and unbalanced sample sizes per cohort, this observation should be interpreted with caution.

Hepatic impairment had no effect on the cabazitaxel unbound fraction with a low free fraction estimated across all cohorts (5.6–6.6%). These results are consistent with the high binding of cabazitaxel to total plasma proteins observed ex vivo and in vitro (89–92%) [2, 15, 17].

Even though cabazitaxel is primarily metabolized by CYP3A in the liver, the minimal impact of hepatic impairment on cabazitaxel PK parameters is consistent with a high cabazitaxel clearance driven by hepatic blood flow and is also consistent with the modest effect that repeated ketoconazole (a strong CYP3A inhibitor) administration has on cabazitaxel clearance; in one study, repeated ketoconazole administration resulted in a 20% decrease in cabazitaxel clearance [20]. Data from this study support the use of cabazitaxel in patients with mild or moderate hepatic impairment at reduced doses of 20 and 15 mg/m^2^, respectively, compared with the approved dose of 25 mg/m^2^. Based on this study and in the absence of appropriate data, the use of cabazitaxel is not recommended in patients with severe hepatic impairment. Based on PK data, there was no evidence of a relationship between safety and PK parameters as the lower MTDs could not be justified by higher cabazitaxel exposure in patients with mild or moderate hepatic impairment. However, dose reductions of cabazitaxel in patients with mild or moderate hepatic impairment, and contraindication in patients with severe hepatic impairment, are justified based on safety data. A recent phase III non-inferiority study (PROSELICA) has demonstrated that cabazitaxel administered at a dose of 20 mg/m^2^ maintains at least 50% of the survival benefit observed with the approved 25 mg/m^2^ dose of cabazitaxel versus mitoxantrone in the previous phase III TROPIC study [1], in patients with mCRPC who have received prior docetaxel treatment [21].
